# Chronic Osteomyelitis of Clavicle in a Neonate: Report of Morbid Complication of Adjoining MRSA Abscess

**DOI:** 10.1155/2016/3032518

**Published:** 2016-03-08

**Authors:** Shishir Murugharaj Suranigi, Manoj Joshi, Pascal Noel Deniese, Kanagasabai Rangasamy, Syed Najimudeen, James J. Gnanadoss

**Affiliations:** ^1^Department of Orthopaedics, Pondicherry Institute of Medical Sciences, Pondicherry 605014, India; ^2^Department of Pediatric Surgery, Pondicherry Institute of Medical Sciences, Pondicherry 605014, India

## Abstract

Osteomyelitis of clavicle is rare in neonates. Acute osteomyelitis of clavicle accounts for less than 3% of all osteomyelitis cases. It may occur due to contiguous spread, due to hematogenous spread, or secondary to subclavian catheterization. Chronic osteomyelitis may occur as a complication of residual adjoining abscess due to methicillin resistant staphylococcus aureus (MRSA) sepsis. We report a newborn female with right shoulder abscess that developed chronic clavicular osteomyelitis in follow-up period after drainage. She required multiple drainage procedures and was later successfully managed with bone curettage and debridement. We report this case to highlight that a MRSA abscess may recur due to residual infection from a chronic osteomyelitis sinus. It may be misdiagnosed as hypergranulation tissue of nonhealing wound leading to inappropriate delay in treatment. High index of suspicion, aggressive initial management, and regular follow-up are imperative to prevent this morbid complication.

## 1. Introduction

Chronic osteomyelitis of clavicle is rare in newborn. Diagnosis of osteomyelitis in presence of overlying abscess may be delayed until established discharging sinus is present as radiological signs may appear late. Most common anatomical site of osteomyelitis is metaphysis of long bones [1]. Other bones like mandible and clavicle are rarely involved and reported as anecdotal cases [2, 3]. In clavicle, middle third is the most common site reported [3]. It may commonly occur as a complication of hematogenous spread in sepsis or from adjoining soft tissue pyogenic abscess. Gram-positive cocci or staphylococcus aureus is the most common causative organism reported in osteomyelitis [4, 5]. We present our experience in dealing with a case of neonatal clavicular osteomyelitis, its difficulty in diagnosis due to late radiological changes in the bone, and the high virulence of MRSA resulting in recurrence and complication.

## 2. Case Report

A 2.8 Kg term female, outborn by vaginal delivery, was admitted in NICU at 36 hours of life with indirect hyperbilirubinemia. Total serum bilirubin was 25.1 mg/dL. Intravenous cefotaxime and phototherapy were started. Sepsis screening reports revealed MRSA growth in blood culture sensitive to vancomycin and linezolid. Screening of mother was negative for MRSA. Antibiotic was changed to intravenous vancomycin on day four of life. On day seven of life a tender, fluctuant swelling was seen over right supraclavicular region along with fever. Upper limb movements were restricted at shoulder joint. It was clinically diagnosed as infected hematoma or abscess. Radiographs of the right shoulder and clavicle did not reveal any bony abnormality. Ultrasonography reported echogenic collection in subcutaneous infraclavicular region. Incision and drainage was done and approximately 30 mL thick pus was drained. Pus culture also reported MRSA sensitive to vancomycin. She was treated with vancomycin for two weeks. Repeat blood culture after drainage showed no growth. But repeat pus culture swab showed scanty MRSA growth. She was continued on oral linezolid for four weeks.

**Figure 1:**
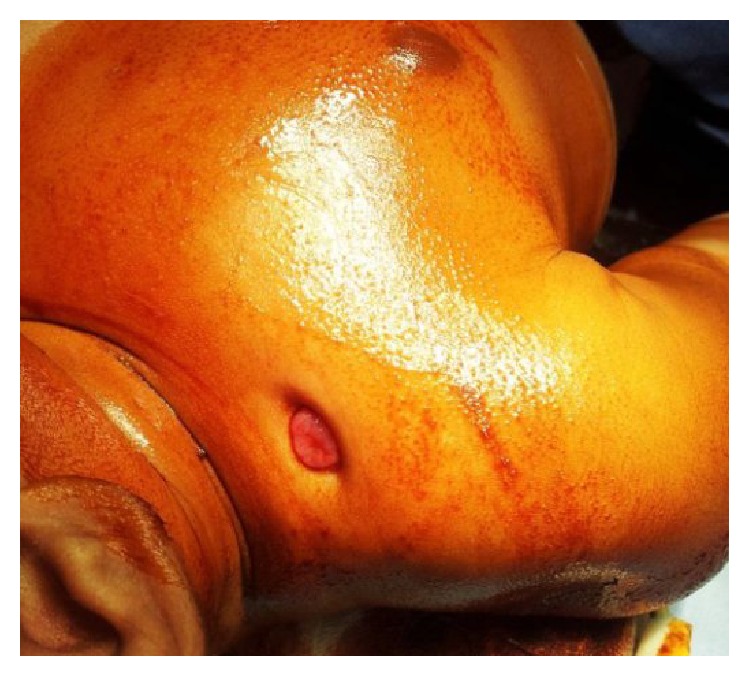
Clinical photograph of the right shoulder showing chronic discharging sinus over the clavicle.

**Figure 2:**
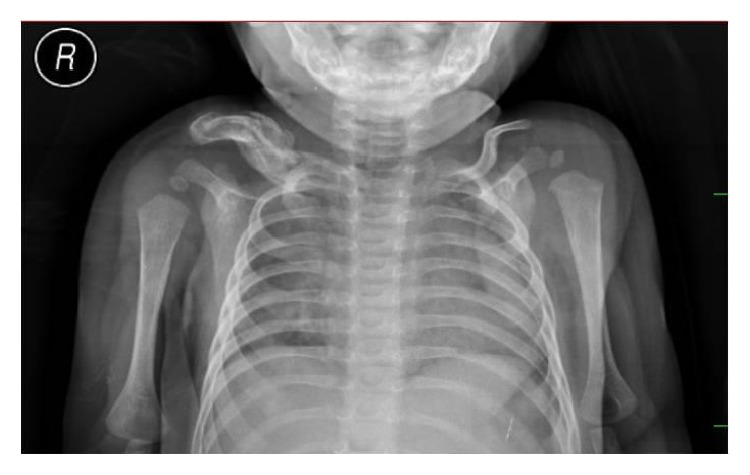
Radiograph showing extensive active osteomyelitis of middle third clavicle with sequestrum and involucrum.

**Figure 3:**
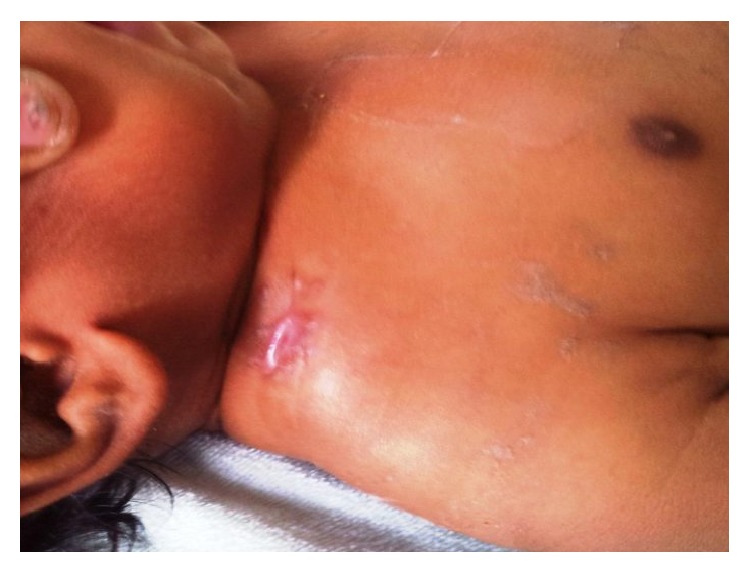
Clinical photograph of the right shoulder showing wound healed by secondary intention.

**Figure 4:**
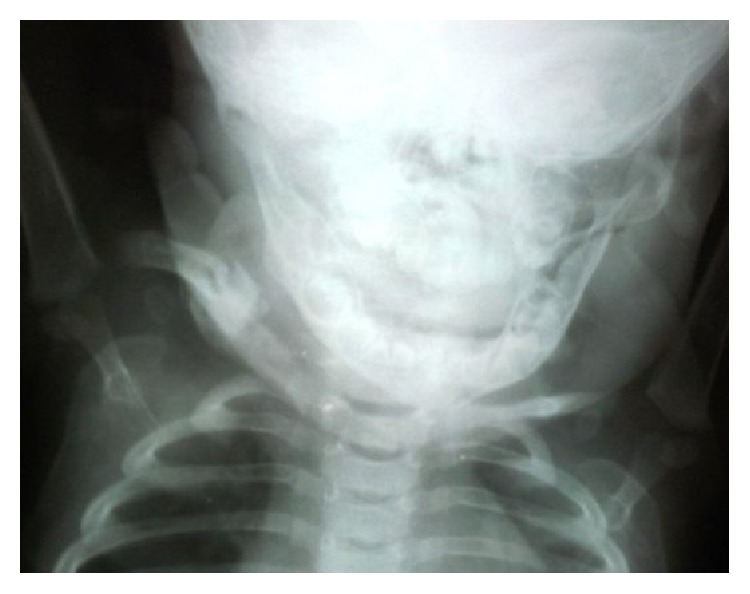
Radiograph six months after operation showing healed osteomyelitis in midshaft of clavicle.

The wound healed initially and she was discharged on day 18 of life. But after one week, at day 25 of life, there was recurrence of abscess at the same site. A repeat drainage was done at a different hospital. The wound healing was incomplete. Subsequently she was treated with copper sulphate application over the discharging sinus for 4 weeks. She was than referred to us at three months of age with chronic discharging sinus (Figure 1). Radiographs of right shoulder revealed extensive osteomyelitis of clavicle (Figure 2). Wound exploration, curettage, saucerization, and debridement with sinusectomy were done. Wound was primarily closed. On the third day, she again developed fluctuant swelling. Sutures were opened and blood mixed pus was allowed to drain. Aerobic and anaerobic cultures were sent. Pus culture again reported MRSA sensitive to vancomycin and linezolid. A course of intravenous vancomycin for 21 days was given followed by oral linezolid for one month. Wound was irrigated with saline and gentamicin which was also specific to MRSA. The discharge gradually subsided and wound healed by secondary intention (Figure 3). Repeat ultrasonography showed no collection in soft tissue and last follow-up radiograph after six months showed healed osteomyelitis in midshaft of clavicle (Figure 4).

## 3. Discussion

Acute osteomyelitis of clavicle is rare and accounts for less than 3% of all cases of osteomyelitis [4]. In a series of 38 children with osteomyelitis Mah et al. reported 2.6% incidence of clavicular involvement [6]. In a case series of 139 children, Dietz et al. reported 4.31% incidence of chronic clavicular osteomyelitis [7]. MRSA is an established nosocomial infection and most common pathogen reported for this rare entity. It is endemic in most of the hospitals and reportedly accounts for 40–60% of all nosocomial staphylococcus infection [8].

Neonates may get nosocomial infection through peripheral or central intravenous cannula site or infection may be community acquired [8]. The onset of nosocomial infections traditionally occurs 48 hours after hospital admission. Hematogenous spread complicates to sepsis, soft tissue abscess, or osteomyelitis of long bones. Local symptoms in acute osteomyelitis may appear late and include swelling over the affected area. Although MRSA is a typical nosocomial pathogen, it is more probable that the soft tissue abscess in our case was due to hematogenous dissemination of MRSA sepsis, for which the infant was admitted to the hospital at 36 hours of life.

Periosteal reaction, lytic areas surrounded by cortical thickening, and swelling of adjacent soft tissue are common radiological findings of osteomyelitis [2]. However, early signs may not be evident in radiographs until one week to 14 days [9]. Ultrasonography is an additional and useful method to diagnose and assess progress of osteomyelitis and its complications. Deep soft tissue swelling is the earliest sign of acute osteomyelitis followed by periosteal elevation and thin layer of subperiosteal abscess [6], but a normal ultrasonography does not rule out osteomyelitis. Computerized tomography scan (CT scan) is more sensitive for detecting early osteomyelitis [2]. Gadolinium enhanced magnetic resonance imaging (MRI) is currently gold standard in diagnosing early osteomyelitis and complicating abscess along with extraosseous soft tissue infection [10]. In our case radiographs and ultrasonography were normal in the initial stages of the disease. In follow-up period it showed extensive cortical erosion of bone, periosteal reaction, and minimal soft tissue collection as evidence of chronic osteomyelitis. These sequences of events suggest that osteomyelitis of clavicle was not present earlier and developed later as complication of residual adjoining MRSA infection or possibly it was not detected by radiographs and ultrasonography in early phase of the disease process. Ultrasonography was more useful to us in follow-up period in assessing residual soft tissue collection and analyzing progress of healing following surgery. CT scan and MRI were not done in our case.

The goal of treatment in neonatal osteomyelitis is eradication of bone infection. This can be achieved by culture specific antibiotics for adequate duration and surgical debridement with adequate curettage. Vancomycin is considered as a drug of choice for MRSA bacteremia. But incidences of treatment failure have been observed [2]. So, there is increasing trend towards use of combination therapy. However, guidelines for duration of antibiotic treatment are not clear and treatment has to be individualized [10].

An aggressive and extensive debridement should be done including that of necrotic bone and surrounding soft tissue in order to prevent the local recurrence and reduce the chances of sepsis. Our patient was initially treated with intravenous vancomycin for two weeks followed by oral linezolid along with drainage of pus. But this treatment appeared ineffective in clearing MRSA completely and complication of osteomyelitis developed. We speculate that it is probably due to lower bone concentration of vancomycin and higher minimum inhibitory concentration (MIC) of vancomycin which was possibly not achieved in our case. In her readmission, after bone curettage, she responded to vancomycin for 21 days along with local irrigation of culture specific gentamicin followed by oral linezolid for one month with no recurrence.

To conclude, we emphasize on the fact that chronic clavicular osteomyelitis is rare in neonates and may happen as a complication of residual adjoining MRSA soft tissue abscess. Radiological signs may not appear in early phase, so high index of suspicion and regular follow-up are necessary to prevent this complication. Guidelines for duration of combination therapy with antibiotics are needed to avoid treatment failure.
